# Untrained Chimpanzees *(Pan troglodytes schweinfurthii)* Fail to Imitate Novel Actions

**DOI:** 10.1371/journal.pone.0041548

**Published:** 2012-08-08

**Authors:** Claudio Tennie, Josep Call, Michael Tomasello

**Affiliations:** Department of Developmental and Comparative Psychology, Max Planck Institute for Evolutionary Anthropology, Leipzig, Germany; Università di Parma, Italy

## Abstract

**Background:**

Social learning research in apes has focused on social learning in the technical (problem solving) domain - an approach that confounds action and physical information. Successful subjects in such studies may have been able to perform target actions not as a result of imitation learning but because they had learnt some technical aspect, for example, copying the movements of an apparatus (i.e., different forms of emulation learning).

**Methods:**

Here we present data on action copying by non-enculturated and untrained chimpanzees when physical information is removed from demonstrations. To date, only one such study (on gesture copying in a begging context) has been conducted – with negative results. Here we have improved this methodology and have also added non-begging test situations (a possible confound of the earlier study). Both familiar and novel actions were used as targets. Prior to testing, a trained conspecific demonstrator was rewarded for performing target actions in view of observers. All but one of the tested chimpanzees already failed to copy familiar actions. When retested with a novel target action, also the previously successful subject failed to copy – and he did so across several contexts.

**Conclusion:**

Chimpanzees do not seem to copy novel actions, and only some ever copy familiar ones. Due to our having tested only non-enculturated and untrained chimpanzees, the performance of our test subjects speak more than most other studies of the general (dis-)ability of chimpanzees to copy actions, and especially novel actions.

## Introduction

In human cultural life there are only a few *right* ways of doing something. These restrictions can be due to social reasons, i.e. norms [1]; or due to physical reasons, e.g. due to the constraints of ever more complex technological demands [2]. On the flip side, this means that there are often sheer endless ways of doing something incorrectly (rendering the behaviour socially or technically non-functional – or less efficient). This is most clearly illustrated in language – which crucially depends on arbitrary, but conventional (i.e.: standardized/copied) utterances. The same logic often applies to actions (most clearly exemplified in sign language; but also in dance routines, rituals, conventions etc.) – including copying the operation of otherwise *cognitively opaque* tools [3], [4]. Humans must thus be able to imitate in various ways in order to blend into their surrounding culture – and make use of it – and indeed humans start to imitate when they are very young, starting from twelve months of age [5], [6].

Within the problem solving domain (common to many species), many types of social learning can be potentially advantageous behavioural acquisition mechanisms: they all can reduce the cost to an individual of trial-and-error learning, or when insight learning is lacking [7], [8]. As would thus be expected, social learning in general appears in a wide range of species (for a current overview see [9]). But this does not mean that imitative abilities (roughly: action copying skills) are useful for all of these species. As we have argued elsewhere, even for great apes, non-imitative mechanisms may suffice and there may thus be no evolutionary (or even ontogenetic) pressure for developing, maintaining or extending imitative abilities [2]. In humans, due to cultural histories that have produced cognitively opaque and/or arbitrary solutions, trial-and-error-learning and insight learning are very often not valid alternatives (anymore) to producing those solutions without copying them [10]. And so, human social learning is not just a more cost-effective way of learning to solve problems that one could have found on one’s own (though it can be), rather it is key to be able to use and participate in modern human culture (and this argument encompasses cultural accumulation and cultural intelligence [2], [11], [12]).

Imitation can transmit behavioural variants relatively intact, and it is for this reason that it underlies the cumulative character of human culture [5]. This is the first step to preservation and improvement of traits [2], resulting in cumulative culture. Eventually culture will have reached a point in which only imitation enables one to blend into his or her surrounding culture. The reason is that cumulative culture leads over time to behavioural strings that can no longer be re-invented on one’s own (they have become too arbitrary/complex and therefore too *improbable* for independent individual discovery). Thus, imitation is necessary for cumulative culture.

It is also worth noting that here we examine the transmission aspect of cumulative culture (arguing that imitation is a particularly powerful way of transmission). Thus, here we do not attempt to examine the innovation aspect of cumulative culture – including potential impacts of previously acquired behaviors (be they acquired socially or individually) on later innovations. Of course, in doing so we do not deny the importance of these questions.

Recently, the idea that imitation is a necessary component of cumulative culture has come under threat from various sources. Caldwell and colleagues [13] have provided evidence that culture can accumulate in the laboratory even when imitative learning can be excluded. On a more theoretical level, Heyes [14] also argued that cumulative culture can come about by other mechanisms than imitation. Thus, the distilled argument is as follows: it is not *a priori* clear that imitation should take precedence in allowing accumulation of culture. Other mechanism, as long as they are precise enough, may allow cultural accumulation as well. This remains a theoretical possibility, which in our view still awaits empirical evidence. Caldwell et al.’s elegant study, in our view, does not fully address this issue because their task cannot be assumed novel to the participants, and thus mere triggering of behaviour patterns learnt earlier in life (possibly via imitation) could have resulted in the findings obtained. But more importantly, as already discussed above, much of human cumulative culture is purely action based, and action copying is best adjusted to pass these types of cultures on (e.g., action based rituals, dancing, sign language etc.).

In addition to being helpful in learning to cope with a cumulated culture of solutions to physical problems as well as a cumulated culture of social conventions (which often, though not always, solve social problems), humans imitate others actions also for immediate social reasons [15] and when these actions are not (or not yet) part of accumulated conventions. Such immediate social imitation may be due to a human need to appear to *be like others* in order to establish and maintain social relations [16]. Our study thus also provides a comparative aspect to this kind of imitation.

Controversy remains over whether apes are able and/or motivated to replicate a particular behaviour exactly (i.e., to copy actions; see [17], [18], [19]). Recent reviews show that there is now little dispute that apes are less inclined to copy actions than humans are [2], [20], however some researchers go even further, claiming that apes may very rarely (if ever) copy actions [17]. There may be several reasons for the apes’ failure to copy actions. Apes might perhaps lack the skill or the motivation to do so – or both (for a review of the evidence, see [2]). Evidence for this view is however mostly based on indirect tests that hint at a lack of action copying, rather than at testing action copying itself.

The current methodological standard of social learning research in apes, which is based on object performance (so-called two-action tasks), does little to help settle this question [2]. Two-action tasks use puzzle boxes (usually with food rewards inside) which can be opened by a demonstrator in more than one way (typically two – hence the term). Yet, problems arise on several fronts: 1. by using an object-based methodology, the typical two-action experiments fail to differentiate between several different types of social learning (especially between copying results or copying actions, see discussions in [17], [21]), unless the methodology is extended by adding somewhat unnatural control conditions (which may themselves be a confound [18]) 2. The actions needed to manipulate two-action tasks are often familiar as well as trivial to subjects in which case one cannot in principle differentiate between the copying of familiar or novel actions and which often results in high baseline occurrences of each action method. A notable exception may be a study by Whiten et al. [22], where subjects did not detect solutions on their own in a baseline condition (though order effects cannot be excluded). But we would like to note that such a state of affairs may be subject to change. For example, in another study by Whiten et al. [23], the same pattern appeared for a similar task (the so-called Pan-Pipe apparatus), in that a baseline condition with a small number of participants showed no success in naïve individuals. A later study, however, showed that both techniques can come about in individuals that had not seen these techniques [24].

Two-action tasks examine only one particular aspect of culture: transmission. They usually do not examine certain features of cumulative culture, such as increase of efficiency and that (human) culture can accumulate complexity (in the sense that complex designs are protected from loss along their transmission). Here we concentrate on the complexity aspect. Since complexity is difficult to operationalize and define, here we have applied one way around this problem by using one correlated feature of complexity in a cultural sense: improbability. The underlying reason for human culture becoming more improbable in design is its’ underlying evolutionary process with the power to produce divergent design (and evolution is a powerful force in producing such improbable design; e.g., [25]). In this way, culture accumulates to the point at which naïve individuals cannot independently arrive at the same cultural design by learning processes other than imitation (see above). The resulting cultural design will then have become improbable (in terms of spontaneous occurrences without imitation). Please note that here we target action-improbability, and thus use action based tasks. Other tasks may also result in high improbability of solution (see above for potential examples) – but if the method allows for successful learning mechanisms other than action copying to arrive at solutions, then no argument can be made that action copying skills were necessary to solve the task (e.g. Bonnie et al. [26], while referring to action copying, represented a two-location task, and thus this was rather a local enhancement-, not an action copying-, task).

And so, any methodology that sets out to study human-like cultural abilities needs to move beyond mere arbitrariness as exemplified in two-action tasks (i.e. style components that are underdetermined by physical necessity but which can still be quite probable each). Such a study needs to study cultural complexity, measured via improbability. Since the focus of this paper is on action copying, the methodology we have used may be called the *improbable-style* method. According to this method, one demonstrator performs an arbitrary action which would *probably* not be performed by the observers by any other learning mechanism than action copying (i.e., it should not occur outside the demonstration context; and this probability is established through a baseline). If a behavioural correspondence between demonstrators and observers is found and if it exceeds the expected occurrence of target action (as derived from the baseline performance), then some form of action copying (or facilitation) has likely taken place. However, it is important to distinguish between two different types of action copying: copying of novel actions and of familiar actions. While the latter have often been referred to as response facilitation [27], recently Byrne [28], [29] adapted a more general distinction originally made in the vocal imitation literature (i.e., [30]) to differentiate between behavioural acquisition processes based on the relative familiarity of the action. Byrne thus introduces two types of action copying – where in both cases actions are observationally learned in order to be used to achieve certain ends. Where the copied action sequences are novel he speaks of *production learning by imitation*, while when the copied actions are already familiar to observers, he speaks either of *response facilitation* – or, if the actions were familiar, but the context is novel – he speaks of *contextual imitation* [28].

Only in production learning by imitation do observers copy a set of body movements or body orientations that are novel to them – and thus only here do they enlarge their behavioural repertoire (see also [20], [31], [32]). Without such a copying mechanism, there is already a limit to the potential accumulation of innovative behaviour (especially if the innovation involves many action style components [5]). Contextual imitation, i.e., the copying of familiar actions, largely fails in such a book-keeping sense ([27]; but see [33]) because the reproduced actions are not actually copied (and thus not added to the repertoire) – instead observers need only recognize familiar actions in others, and then to trigger those same actions within themselves in new contexts. Since these behaviours (as a whole) already form part of the subjects’ repertoire, their complexity/improbability level is far lower than is the case for novel behaviours, and this inherent restriction severely limits the type of cultures that may develop (though see [33] for a proposal that may unify the two forms of imitation).

Another important factor to consider is enculturation, i.e., whether or not the animal has received extensive human contact (possibly even in the form of extensive training) which may have led to changes in several socio-cognitive domains of subjects (see [34], [35]). Apes who have been raised like human children for most of their life [36] or those who have been raised at least by humans for some prolonged time period [19] and/or who have received extensive human training during their lives, can sometimes show measurable action copying skills (possibly all these are subsumable under the general label enculturated apes; see also recent reviews: [19], [37]). This data could also be taken to suggest that the lack of copying so far found in non-enculturated apes may be due to a lack of motivation rather than a general lack of skill [2]. Yet interestingly, enculturated apes copy more frequently transitive actions (actions that are goal directed, as for example when they are *anchored* to objects, including body parts; see [31]), which may suggest some extra copying power may still be generated due to the apes’ proneness for emulation learning (which is in effect learning about the environment; see review in [37]). Also important is the fact that, while enculturated apes sometimes copy actions, they do so with a general low fidelity as compared to humans (review in [19]).

Byrne and Tanner [19] proposed an intriguing potential explanation for why actions - even by enculturated apes - are only poorly copied. Byrne and Tanner suggest that the currently available evidence on action copying in apes is all best explained by contextual imitation. That is, the tested apes might have matched demonstrated target actions with those actions in their repertoire which “most resembled the demonstrated action” ( = copying of familiar actions). As support for their claim, Byrne and Tanner present action copying data on a nursery-raised gorilla. The actions that were copied by this individual would have been regarded as novel (production imitation) – if the methodological standards of the earlier publications had been applied. Yet, due to favourable circumstances, Byrne and Tanner were able to determine nearly the full repertoire of their case subject prior to the study period – and indeed this analysis showed that none of the actions the subject seemed to have copied had been strictly novel for her. Since no other published claim of action copying in great apes undertook the same kind of detailed analysis, it is entirely possible that no ape ever copied a novel action in the above studies. It is worth noting that the same type of critique has been placed for imitation studies in humans (a view compatible with [38]).

The view that living in a captive environment improves or impairs great ape cognition has adherents on both sides, but we fully agree with Henrich et al.’s [39] view that – ultimately – this question can only be answered empirically. Yet generally, studies with enculturated apes (while certainly important for determining the ontogenetical flexibility and general potential of apes) help little in settling the question of whether wild apes copy actions of any kind. The reason is simply that enculturation by humans does not happen in the wild. Instead, we believe that – with this particular question in mind – it is more ecologically valid to study non-enculturated apes instead.

Here we tested non-enculturated chimpanzees that were born in the wild and who lived in a semi-natural environment on a freshwater island in Africa. We looked at whether these subjects would show evidence for action copying of familiar and/or novel actions in tasks where the demonstrated solution was based solely on action style (i.e., target actions). Sometimes, target actions were demonstrated within a non-begging context (i.e., they were transitive actions towards an unmoving apparatus), whereas in other cases target actions were directed towards a human in a social context (begging, i.e., here actions were gestural and intransitive – directed towards a human with the goal of receiving food rewards from this human). Observer chimpanzees were given a chance to perform the target actions after demonstrations; for which they received rewards. For each type of target action, baseline conditions established levels of target action occurrence without prior demonstrations.

In general, we adapted some of the basic methodological features of an earlier study by Tomasello et al. [40]. Tomasello and colleagues trained several chimpanzees to perform novel begging gestures towards humans in exchange for food rewards. The trained chimpanzees subsequently demonstrated their newly acquired begging gestures as target actions while naïve conspecifics watched. In Tomasello et al.’s study, observer chimpanzees failed to copy, which may be indicative of a general inability or unwillingness in chimpanzees to copy actions. Yet, this negative finding may also be due to two factors which were not excluded in that particular study: first, target actions were exclusively novel actions (thereby testing for production imitation, which chimpanzees might be unwilling and/or unable to do) and, second, observers were required to learn a new begging gesture – even though they were already able to use (different) begging gestures prior to the study. This *prior-usage* of different begging gestures may have had detrimental effects on the subjects’ motivation and/or ability to learn further begging actions. Indeed, recent studies suggest that chimpanzees are reluctant to learn new solutions to problems for which they have already found a solution (“conservatism”; see [41], [42], [43], [44]), and relatedly, they may also show functional fixedness [45], [46] – though see a recent paper by Dean et al. [47] for a different view.

We improved on the original method of Tomasello et al. [40] in the current study in order to avoid these same criticisms to some degree. In our first study, we tested chimpanzees in a strictly non-begging context in which they had not previously arrived at a solution (we used an apparatus instead of a human to dispense the rewards). Only later did we test subjects within a begging context. Another improvement implemented in our first two studies was that the chosen action was familiar rather than novel to the observers – thereby enabling the observers another route by which to learn (namely, contextual imitation instead of production imitation). For one individual in particular, we added conditions in which we increased the level of complexity – and, for this special subject, eventually we also replicated the original methodology of Tomasello et al. [40]; i.e., using a novel target action in the begging context.

## Materials and Methods

### Study Site and Subjects

Studies 1 and 2 were done in 2006. Studies 3 to 5 were done in 2007. Data collection took place at Ngamba Island Chimpanzee Sanctuary, Lake Victoria, Uganda, which is a sanctuary for chimpanzees (*Pan troglodytes schweinfurthii*) born in the wild who have been rescued from various trade-markets in Uganda and the surrounding countries (http://www.ngambaisland.org).

### Ethics Statement

In accordance with the recommendations of the Weatherall report “The use of non-human primates in research” subjects are allowed to roam freely on the 40 ha island covered with tropical rain forest during the day and spend the night in seven interconnected sleeping rooms (approx. 140 m2) with regular feedings and water ad lib. Subjects voluntarily participated in the study and were never food or water deprived.

No medical, toxicological or neurobiological invasive research is conducted at Ngamba Island. Our research was non-invasive, strictly adhered to the legal requirements of Uganda and was approved and reviewed by the Ugandan Wildlife Authorities (UWA) and the Ugandan National Council for Science and Technology (UNCST). The study was ethically approved by committees of the Max Planck Institute for Evolutionary Anthropology and the Chimpanzee Sanctuary & Wildlife Conservation Trust. Animal husbandry and research comply with the “PASA Primate Veterinary Healthcare Manual” and the “Guidelines for the Treatment of Animals in Behavioral Research and Teaching” of the Association for the Study of Animal Behavior (ASAB).

### Training Procedure

One of us (CT) used clicker training as well as manual shaping to train one male chimpanzee (Mawa; estimated date of birth 1996) to perform the desired target actions prior to our studies. We trained two target actions in succession (different target actions were used for Studies 1 and 2 versus Studies 3 to 5). After each set of studies, we stopped reinforcing the trained actions and thus added extinction phases [48].

We chose Mawa because he was the dominant male over a large number of subjects, and so could perform demonstrations as well as receive the resulting rewards without interference from other, less dominant individuals. Furthermore, he could be moved with ease from one room to another. A male demonstrator was chosen because in the original action copying study by Tomasello et al. [40] all demonstrators were females, and this may have been a factor in causing the negative findings of Tomasello et al. (the same could be said of the trained demonstrations in [47]). There are now numerous studies which included male chimpanzee demonstrators that detected some social learning [18], [46], [49]. Time constraints did not allow us to systematically check for the possible significances of demonstrator sex (or other attributes) in this study – even though we do not deny that these factors may be important (i.e. model-based copying strategies, as described by [50]). In addition, we choose a male, as females are the transferring sex in chimpanzees, yet their cultures seem to persist over time [51].

### General Procedure Across All Studies

All studies had in common that a target action was demonstrated to one or more observers by Mawa where Mawa was rewarded with food for performing these actions. We measured whether we could detect copying of these target actions in observers. Which target action was used and whether or not an apparatus (food dispenser, see below) was involved depended on the exact study. Whether or not there was a baseline condition, the exact appearance of the apparatus and the composition of subjects depended on the exact study as well. The details of each study are explained in the specific method sections below, but an overview of the most important details can also be found in Table 1, Table 2 and Table 3.

**Table 1:** Method overview for Studies 1 to 5.

		Study 1	Study 2	Study 3	Study 4	Study 5	Grand total
Target action		Presenting	Presenting	Chimpanzee praying	Chimpanzee praying	Chimpanzee praying	
Apparatus used?		Yes (Presenting board)	No (Begging study)	No (Begging study)	Yes (Prayer-board)	Yes (Mini-prayer board)	
Full demonstration condition	Baseline trials	5	3	2	0	0	10
	Full demonstration trials	10	6	8	15	15	54
	Target action demonstrations	30	60	80	45	45	260
Baseline condition	Baseline trials	15	9	n.a.	n.a.	n.a.	24
	Target action demonstrations	0	0	n.a.	n.a.	n.a.	0

**Table 2:** Overview of subjects and outcomes of Study 1 and Study 2.

Subject name	Sex	Age in years	Study 1			Study 2	
			Condition	# Target actions- live coding?	# Target actions- reliability trials?	Condition	# Target actions- live coding?
**Demonstrators**							
Mawa	M	10	Demonstrator - Full Model	n.a.	n.a.	Demonstrator - Full Model	n.a.
Sally	F	15	Demonstrator - Baseline	n.a.	n.a.	n.a.	n.a.
Asega	M	8	n.a.	n.a.	n.a.	Demonstrator - Baseline	n.a.
**Subjects**							
Baluku	M	8	Full demonstration	1	8	Full demonstration	10
Indi	M	7	Full demonstration	0	n.a.	Baseline	0
Kalema	M	10	Full demonstration	0	n.a.	Baseline	0
Okech	M	5	Full demonstration	1	n.a.	Full demonstration	0
Pasa	F	7	Full demonstration	0	n.a.	Baseline	0
Nani	F	5	Full demonstration	0	n.a.	Baseline	0
Kazahukire	F	7	Full demonstration	0	n.a.	Baseline	0
Yoyo	F	7	Full demonstration	0	n.a.	Baseline	0
Umutama	M	10	Baseline	0	n.a.	Full demonstration	0
Bwambale	M	7	Baseline	0	n.a.	Full demonstration	0
Umugenzi	M	9	Baseline	0	n.a.	Full demonstration	0
Nakuu	F	5	Baseline	0	n.a.	Full demonstration	0
Namukisa	F	7	Baseline	0	n.a.	Full demonstration	0
Ndyakira	F	7	Baseline	0	n.a.	Not tested	0
Nkumwa	F	10	Baseline	1	0	Baseline	0

**Table 3:** Overview of subjects and outcomes of Study 3, 4 and 5.

Subject name	Sex	Age in years (at the time)	Study 3		Study 4		Study 5	
			Condition	# Target actions- live coding?	Condition	# Target actions- live coding?	Condition	# Target actions- live coding?
Mawa	M	11	Demonstrator - Full Model	n.a.	Demonstrator - Full Model	n.a.	Demonstrator - Full Model	n.a.
Baluku	M	9	Full demonstration	0	Full demonstration	0	Full demonstration	0
Okech	M	6	n.a.	n.a.	n.a.	n.a.	Full demonstration	0
Bwambale	M	8	n.a.	n.a.	n.a.	n.a.	Full demonstration	0

### Food Dispenser

Tomasello et al. [40] already raised the possibility that the apes in their study might not have copied the novel begging gesture because they already used (other) begging gestures: to counter this objection we included a new apparatus condition (Study 1, Study 4 and Study 5; see below) which took the apes outside the food begging context by introducing a context where observers would not have any pre-formed behaviours.

**Figure 1:**
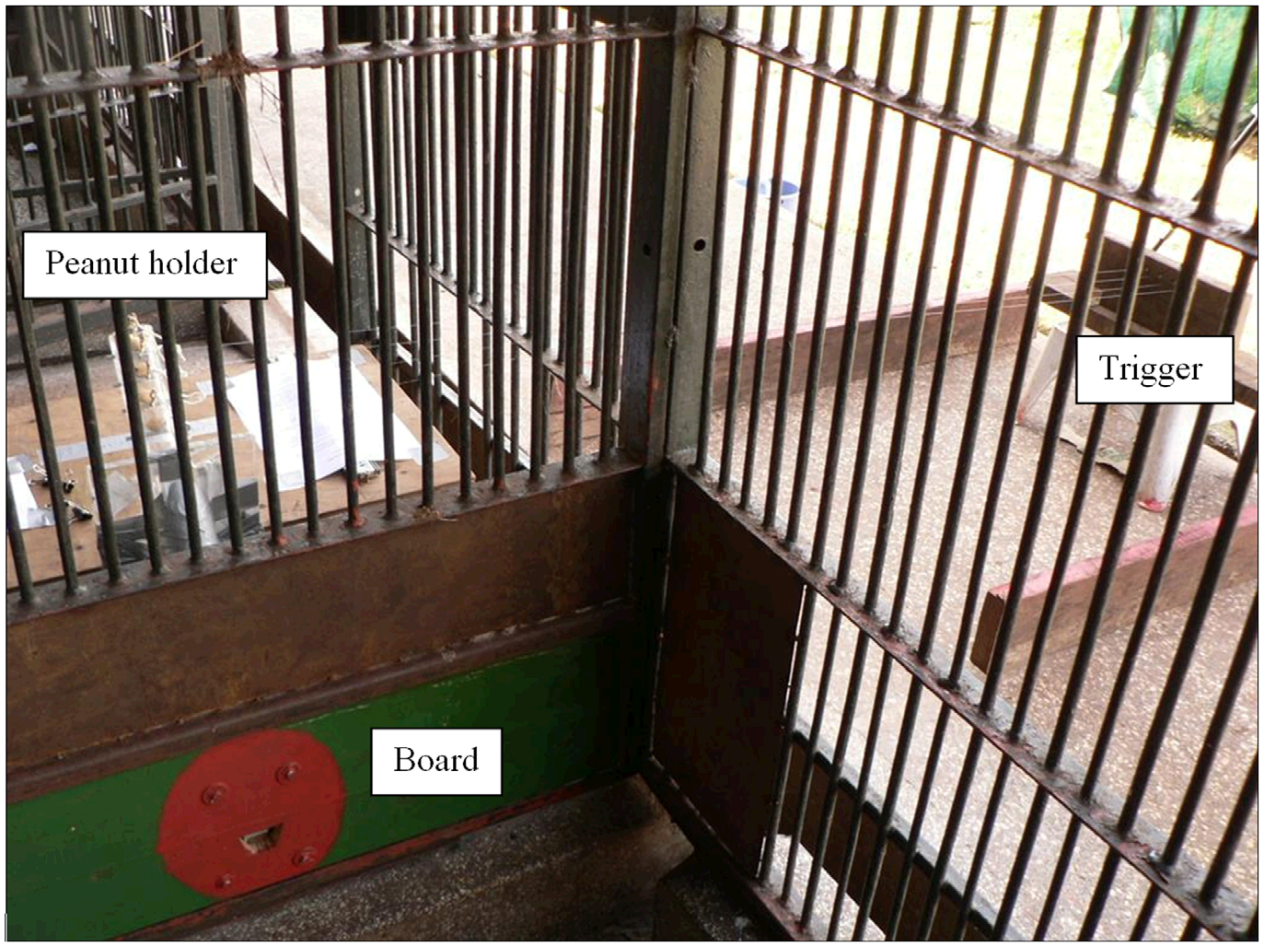
Picture of the food dispenser apparatus, as seen from the ape area. The apparatus was composed of three parts: the board (left bottom), the peanut holder (left middle, behind the mesh) as well as the trigger (right middle).

As the apparatus we devised a remote-controlled food dispenser that would deliver up to four food rewards in succession (see Fig. 1; food rewards were peanuts still in their shells; dispensed one nut at a time); this was so that the apparatus conditions would be kept as technical (i.e., non-begging) as possible. The main experimenter watching the chimpanzees decided if a demonstration had been successful or if a subject showed a close-enough approximation of the target action, and if so, gave a pre-established signal (an inconspicuous sign such as E1 quickly lifting his chin) to another experimenter who remote-controlled the apparatus so that a peanut got released into the reach of the ape. After demonstrations had taken place, the apparatus still contained one last food item that could thus be dispensed to observers without the need to refill after demonstrations.

The food dispenser consisted of three parts (each with their own sub-parts): A board, a peanut holder and a trigger (see Fig. 1; more details on the workings of each part available on request). The board was the only part of the apparatus that was accessible from the ape’s side. It consisted of a painted piece of wood (which changed in appearance between studies, see below for details) with a hole for the released peanut roughly in its centre.

The only visible food was the peanuts in the apparatus. All other food was placed out of the subjects’ view. This setup discouraged subjects from begging either from E1 or E2, and both were also far from the apparatus: in fact, subjects did not beg from E2 or E1. To further ensure that subjects were not distracted during testing, prior to every testing day the whole room was cleaned and any loose items and food pieces were removed.

### Extra Reliability Tests (Apparatus Studies Only)

We added the possibility of extra tests that tested for reliable target action performance in all studies that used an apparatus (i.e., all studies except Studies 2 and 3). The reason is as follows. It was a design feature of all studies that E1 had to observe and decide there and then whether subjects had performed the demonstrated target action. If he judged they had, a reward was made available for this subject.

Due to the nature of our experiment E1’s live judgement could be deemed to have interfered with the course of the study (i.e., E1 chose either to dispense or withhold a reward depending on his immediate conviction as to whether or not the target action had been demonstrated by the subject). The apparatus studies allowed only for one chimpanzee to show target behaviour once during the test trial (since the apparatus only contained one last reward item after demonstrations had happened). In these studies, therefore, we had to devise some additional means of verifying whether the target action occurred by learning and not by chance. Thus, in the studies that used an apparatus, and after the first occurrence of the behaviour was detected by E1, we tested for re-occurrence of the target action in later trials (but not if target action occurred only in the first trials of the day, due to the fact that later trials themselves acted as verification trials). A further criterion was that when the reward was released for subjects they had to collect it (as a minimal criterion for an understanding of the performance of the target action). If they failed to do so, then this subject did not enter the extra reliability test. In summary, if the target actions appeared only in later trials (i.e., in trial two and/or trial three), and if the subject retrieved the reward, the extra reliability test was administered to the subject. Subjects were then tested alone. Such verification tests were given on two separate days, lasted 10 minutes each and in each session the subject could retrieve a maximum of 4 rewards (i.e., a total of 8 rewards across both sessions). As mentioned, we did not have to perform these tests in the social context studies (Studies 2 and 3), as these were not restricted by the one remaining food item in the food dispenser apparatus. Instead, in the social context studies we simply allowed more target actions to occur during trials since here it was the human experimenter that could give out rewards as needed (and there we allowed ten rewarded target actions).

Successful subjects that reliably showed the target action were then excluded from the respective study so as not to interfere with the others subjects’ behaviour (i.e., so that they would not collect all rewards for themselves, potentially leaving other subjects no opportunity to perform).

### Coding and Data Analysis

All trials were videotaped using two video cameras which filmed the area close to the rewards from two different perspectives. During the tests, E1 coded live whether or not the target actions had occurred in the subjects (and if so, either provided a reward directly or had E2 provide a reward). To assess inter-observer reliability a naïve coder coded all trials from videotapes for which E1 had judged a target action to have occurred. This type of reliability was appropriate since the way that rewards were distributed to subjects had already been contingent on the prior judgements of E1– and this was outside the scope of later manipulation (i.e., the nature of tests such as Tomasello et al.’s [40], means that necessarily E1’ judgements interfere with the course of the testing). Overall, and across all studies, E1 deemed three subjects’ performances close enough to count as target actions. However, the independent coder only deemed the performance (across all occurring instances) of one subject appropriate: a male (Baluku, eight years old). Furthermore the independent coder found evidence for only for one type of target action: the familiar target action (presenting). Thus, using this measure, we found reliable evidence for only one type of target action (presenting) performed by only one chimpanzee (Baluku).

## Study 1 Presenting-Board

### Methods

#### Subjects

15 chimpanzees participated in Study 1 (eight female, seven male; mean age = 7.4 years; see table 2).

#### Materials

We used the food dispensing apparatus, ‘the presenting board’, described above. In Study 1, the exchangeable wooden presenting-board apparatus which was facing the apes was 27 cm high x 93 cm broad and was painted dark green with a red circle painted around the reward collection hole. This red circle was 25 cm in diameter, the reward hole was 30 cm above ground level and positioned in the middle of the board. The reward hole itself was five cm long and four cm high and was simply cut from the wood.

#### General Procedure

**Figure 2:**
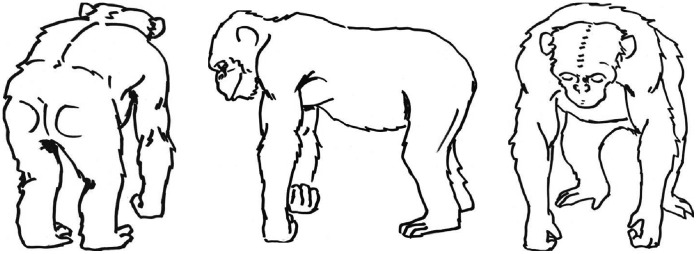
Schematic drawings of the target action presenting; used in Study 1 and 2.

Study 1 was split into two conditions: a full demonstration and a baseline condition (see below). For Study 1, we trained Mawa (male, 10 years old) to perform presenting (see Fig. 2): here Mawa was trained to turn around, stand on all fours and then press his back against our apparatus (the presenting-board). We split subjects randomly into two groups matching both groups as closely as possible for age and sex. Group A (n = 8; mean age = 7 years; four females; four males; see table 2) was given the full demonstration condition, which meant that they were given demonstrations of the target action by Mawa. Group B (n = 7; mean age = 7.9 years; four females; three males; see table 2) were put into the baseline condition and received no demonstrations of the target action. During the demonstration condition, E1 scored live whether a given subject had observed at least one demonstration per testing day (defined as having the head directed towards the demonstration during the demonstration). *Ad libitum* sampling was used, and the results of the live-coding were immediately noted on coding sheets. On the first day of testing, both conditions began with the whole group together in the testing room. On day one, three live coders were present. For that particular day it could be well established whether a given subject had seen at least one demonstration. However, because this number of live coders could not be maintained throughout the study, we had to split the groups into subgroups for the remaining test days. Thus, on all subsequent test days, we split each group into three sub-groups which were then tested separately (the composition of these sub-groups was randomly determined from the pool of subjects in this condition each day before testing started). This way, each condition group was divided into either pairs or triplets for a given test day. Each day, and in both conditions, subjects entered the testing room and were then given several minutes to show the target actions themselves without having seen any demonstrations yet – at least on that day (i.e., a general baseline was established in both conditions, and repeated each day). On day one, each trial, including the first baseline trial, lasted ten minutes. Due to the splitting of groups on subsequent days and the necessary extra time needed for testing, this had to be reduced to five minutes per trial in each condition. Each group received 3 trials per day (consisting of one baseline trial and two experimental trials in the full demonstration condition, and three baseline trials in the baseline condition). There were five testing days for each condition, resulting in 15 trials overall (of which ten trials were experimental trials in the full demonstration condition).

#### Full demonstration condition

In the full demonstration condition, on each testing day, subjects were first tested in a baseline trial. Then, before trials two and three, the trained demonstrator (Mawa) entered the testing room. He provided three demonstrations (i.e., rewarded presenting) before the beginning of each experimental trial (altogether six demonstrations per testing day, resulting in a maximum of 30 observable demonstrations per subject across the course of Study 1). Mawa received no verbal commands – except that his name was occasionally called to get him to attend and thus perform the demonstrations. Once Mawa had left the testing room, the trial began. See online supplementary material for a screenshot (Figure S1) of the full demonstration condition of Study 1 (taken during the demonstrations).

#### Baseline condition

The baseline condition was basically the same as the full demonstration condition, with one difference: an untrained chimpanzee entered the testing room prior to trials two and three in order to present *pseudo-demonstrations* – controlling for factors such as local and stimulus enhancement. Crucially, instead of demonstrating the target action, the pseudo-demonstrator performed no particular prescribed action, while she still was provided with the same three rewards. In Study 1, this chimpanzee was Sally, an adult and dominant female (15 years old; we used a female because at that time the only other male with the required characteristics of dominance and moveability (Asega; eight years old) was unavailable). See online supplementary material for a screenshot (Figure S2) of the baseline condition of Study 1 (taken during the pseudo-demonstrations).

### Results

See table 2 for an overview of the main results.

#### Observation level

In general, subjects were attentive to demonstrations. In the full demonstration condition, every subject watched at least one demonstration on each testing day, as established through live coding.

#### Baseline condition

One subject (Nkumwa, female, ten years old) in the baseline condition twice showed (live-judged) signs of target action approximations: once on the second day of testing (in her third trial) and once on the third day of testing (again in her third trial). In neither case, however, did Nkumwa’s actions closely match the demonstrator’s actions to be judged as matching the presenting action (as also later judged by the reliability coder, see above). Nkumwa’s approach to the board first involved facing the board (which was the usual approach also of others), then turning around so that her back touched the board, while either a finger was inserted into the reward hole or while she scratched the board around the hole with her fingernails (and she also kicked it backwardly with a foot). For example, this meant that Nkumwa was not standing on all four when she was touching the board with her back. Due to all these mismatches with the target requirements, Nkumwa was not rewarded on this first occasion. However, on the second occasion (i.e., on testing day 3) E1 decided to reward her, even though her actions did not change from the first occasion (E1 felt that Nkumwa was performing at least part of the required body configuration – and especially because by then she had done so repeatedly). This meant that Nkumwa fulfilled the criterion to receive extra reliability test. But in these additional testing sessions she failed to perform even approximations of the target action.

#### Full demonstration condition

**Figure 3:**
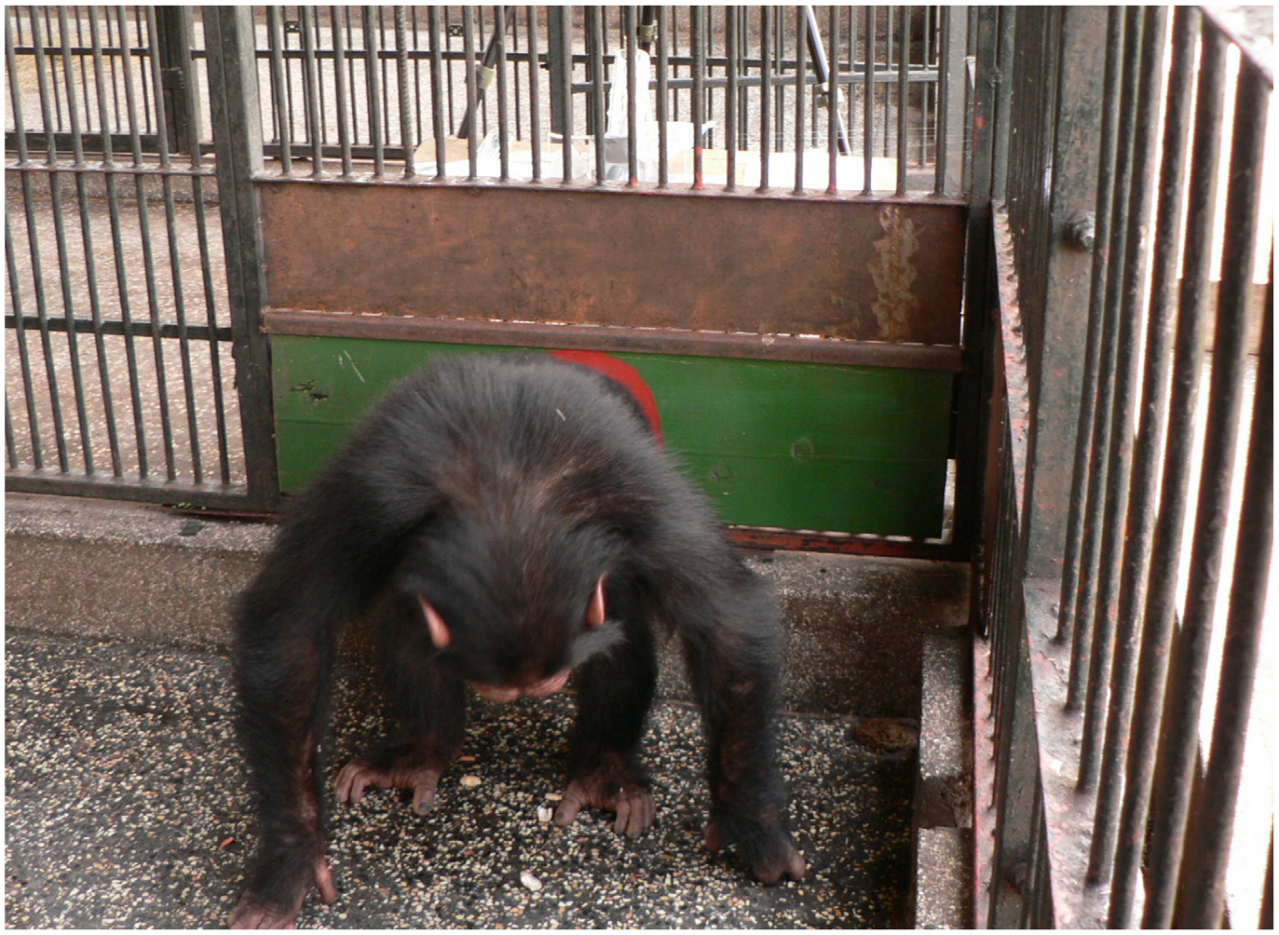
Picture of Baluku’s performance of the target action (i.e., presenting) in Study 1.

Two subjects were live-judged to have performed approximations of the target actions in the full demonstration condition: Okech (male; five years old) and Baluku (male, eight years old). Okech was judged by E1 to have performed the target action in his first trial of day two (while Okech had not seen any demonstrations on that day prior to that particular trial, he had already seen six demonstrations on test day one). Okech performed two target action approximations in this trial. While his first approach was not rewarded by E1 (since it looked like a very distant approximation), E1 rewarded Okech on the second occasion, for this attempt seemed a closer approximation to the target action. In the first instance, Okech had one arm on the mesh while simply swinging around. In the second instance Okech had both arms on the ground – however on this occasion he lowered his torso to the floor and gathered a piece of debris from the floor with his mouth, in an apparently playful manner. This movement lifted his behind into the air where it then – seemingly unintentionally – may have touched the board. Once the reward was released, Okech made no attempts to collect it; in fact he seemed oblivious to it. In line with a chance interpretation, Okech did not wait or look for the reward (instead he leisurely left the area and another male chimpanzee (Indie; seven years old) collected the reward instead). This happened on Okech’s first trial on that testing day and he did not repeat the performance nor did he become better in later trials; in line with our procedure we therefore refrained from testing him in the reliability test sessions.A very different behaviour was found in another male, Baluku (eight years old). Baluku already began to show very close approximations of the target action by the second trial of the first testing day (i.e., immediately after he had seen the first three demonstrations of the target action). As judged both by live-coding as well as by the reliability coder (see above), Baluku’s behavioural style was immediately a perfect match of the demonstrated target action. While performing the target action (and this is true even of the first occasion), Baluku watched the presenting-board closely and looked back (seemingly) to determine the exact moment that the reward would be released – which, based on E1’s live-judgement, he received (and took). As he fulfilled all the necessary criteria, Baluku was then tested in the extra reliability sessions, in which he collected all eight of eight possible rewards by showing perfect target action in each case (see Fig.3. for Baluku’s performance; see also Supporting Information for a video (Video S1) of his performance). Also here Baluku watched the board whilst performing the target actions. And once the reward was released, he quickly turned round to gather it, and then immediately repeated the target action. If the rewards did not fall immediately after performing the target action (the peanut dispenser stopped dispensing peanuts after being emptied), he increased his efforts by pressing his back against the presenting-board more vehemently and in quick succession, while glancing back at the board. Baluku was thus excluded from further testing days and judged to be a reliable performer of the target action.

### Conclusions

Altogether, three subjects were judged there and then by E1 to have performed the target action in Study 1, but only one subject (Baluku, male, eight years old; in the experimental condition) showed evidence for having actually copied the target action. Not only did Baluku look back repeatedly whilst performing the target action, expecting rewards to follow, but he also was known prior to this study for being a good candidate for action copying (earlier anecdotal evidence: [52]). Most importantly, however, he was the only subject to have reliably used the target action (as measured by the extra reliability test and the reliability coder). This is despite the fact that he had not previously shown this behaviour towards anything other than conspecifics (personal observation; please note that the presenting action was selected as a target action precisely because of its scarcity in situations like ours).

We believe that Study 1 clearly established that Baluku had copied the target action. While the baseline probability of our chosen target action might not have been zero (as evidenced by some approximations of the target action in the baseline condition), reliable target actions only occurred in Baluku, and only after he had seen demonstrations of it. Also, because the target action (presenting) used is a familiar action to chimpanzees [53], a genuine zero baseline should not even be expected.

Baluku’s pressing his back against the board was a very conspicuous behaviour – due to its repetitiveness and forcefulness. Potentially this could indicate that, even though we designed our task as a non-instrumental task (where only action styles have got an effect), it was regarded by Baluku as something of an instrumental task after all (i.e. “the board required pressing force to deliver rewards”). While this is possible, it does not explain why Baluku would not simply use his hands to press the board (a logic that resembles that used in [54] – though these authors used enculturated apes for testing).

One potential criticism raised against regarding Baluku as an action copier here is that he was rewarded for performing the target action. Thus, one might expect him to continue to perform these target actions, because of this reinforcement and independent of any action copying. We acknowledge that we cannot fully discount this possibility, but we would like to note that chimpanzees are generally performing very poorly if they are rewarded for what appears to be arbitrary behaviours (e.g [55]) and which would seem inconsistent with what would be one-trial learning here.

Tomasello et al. [40] concluded that chimpanzees would not copy novel actions in a social context. We designed Study 2 to determine whether chimpanzees are able to copy a familiar action in a social (begging) context. Study 2 used the same familiar target action as was used in Study 1, but it was now presented in a social context: here we had our demonstrator (Mawa) present directly towards the human experimenter in a begging context (for which Mawa was rewarded). Again, we included a baseline condition for comparisons. In Study 2, food rewards were given manually, instead of (seemingly) automatically by a machine (as in Study 1). In sum, Study 2 can be seen as a close methodological match to the original gesture copying study by Tomasello et al. [40], except that we used a male demonstrator (Mawa) and tested for a familiar target action instead of a novel one.

## Study 2 Presenting as Begging

### Methods

#### Subjects

For Study 2 we mostly switched the groups of Study 1, so that the former full demonstration condition now became the baseline condition, while the former baseline condition became the full demonstration condition. However, some exceptions were made. We again placed both Baluku and Okech in the experimental condition, as they were the only subjects to perform the target action in the experimental condition of Study 1 (Okech only had shown approximations of the target action). Again we placed Nkumwa, who showed approximations of the target action in the baseline condition of Study 1 into the baseline condition of Study 2 (in order to see whether she would again perform approximations of the target action – still without having seen demonstrations of it). Furthermore, one female subject (Ndyakira; seven years old) had to be excluded from the study because she refused to participate further. The full demonstration condition therefore consisted of seven subjects (mean age = 7.3 years; two females, five males; see table 2) as did the baseline condition (mean age = 7.6 years; five females, two males).

#### General procedure

For Study 2, we trained Mawa to perform presenting (see Fig. 2) in a different context from Study 1: here Mawa was trained to turn around, stand on all fours and then press his back against the mesh of the cage in E1’s direction. Thus, Study 2 resembled Study 1 in all respects except the following: there was no E2, no apparatus was used and food rewards were given manually by E1. Two major changes to the general procedure had to be made because of time pressures. First, all subjects had to be tested (in all trials) as a complete group. Yet, as the workload for E1 in Study 2 was comparatively lower (i.e., an apparatus was no longer required and no E2 had to be signalled to), E1 was able to live-code the observation level of subjects in Study 2 in the same way as was done in Study 1 (where groups were split). Second, we only had time for three testing days in Study 2, which resulted in nine test trials per condition (three baseline trials and six experimental trials in the full demonstration condition, and nine baseline trials in the baseline condition). However, even though fewer trials were performed, overall more demonstrations were given to the subjects in Study 2 than in Study 1 (ten demonstrations before each experimental trial in Study 2). All test trials lasted ten minutes. Unlike Study 1, subjects did not need to be given an extra reliability test if they performed the target actions during trials since in Study 2 there was no food dispensing apparatus to restrict the number of rewards. Instead, subjects who showed the target actions in Study 2 were given the chance to perform a maximum of ten target actions during their trials (being rewarded in each case manually by E1). Any such subject was then excluded from further testing (see also above).

#### Full demonstration condition

In the full demonstration condition, each test day started with one baseline trial, followed by two experimental trials. For demonstrations, and before the experimental trials started, our demonstrator (Mawa) entered the research room with all subjects present from the adjacent room. Demonstrations were as follows: Mawa turned around and presented to E1, for which he was rewarded every time (i.e., he turned around again in order to collect the reward, 1/6th of a banana, from E’s hand). Mawa was required to perform ten such demonstrations before he was moved out and the experimental trial began (thus, there were 60 demonstrations across all test days). During the trials and before each demonstration, E1 held 1/6th of a banana in his slightly extended hands, but out of reach to subjects (about one meter from the mesh).

#### Baseline condition

The baseline condition resembled the full demonstration condition in all respects except the following. Instead of using Mawa as a demonstrator, another dominant male (Asega; eight years old) received ten 1/6th pieces of banana prior to trials two and three, on each testing day. In order that Asega did not demonstrate the *wrong* actions (a potential confound of the baseline condition of Study 1) he remained isolated in the adjacent room during these pseudo-demonstrations and could not be observed by the other subjects. Subjects could only infer from E1 calling Asega’s name and from E1 passing rewards into the next room that another chimpanzee potentially got them, but in any case they still had to come up with their own begging actions, uninfluenced by any direct observations. Rewards to the pseudo-demonstrator in the baseline condition were not dependent on performance: Asega was simply called, E1 waited a few seconds, and then Asega was given a reward (as a close approximation of reward-giving in the full demonstration condition).

### Results

#### Observation level

In general, subjects were again attentive to demonstrations. In the full demonstration condition, each subject watched at least one demonstration on every testing day.

#### Baseline condition

No subject in any trial in the baseline condition ever performed the target action. Instead, subjects attempted to use their usual begging gestures (e.g. extending their arms towards E).

#### Full demonstration condition

No subject produced the target action in the initial baseline trial of the full demonstration condition. However, in trial two of the full demonstration condition (that is: after the very first demonstrations in that condition), Baluku performed ten target actions in quick succession (and was rewarded each time). Afterwards, Baluku was released from the research room and was not tested again as he had reliably shown the target action. No subject other than Baluku showed even an approximation of the target action in this or other trials. Instead, these subjects attempted to use their usual begging gestures (e.g. extending their arms towards E).

### Conclusions

The primary purpose of Study 2 was to examine whether chimpanzees possess the ability to copy familiar actions (contextual imitation) in a social context. The results indicate that this can indeed be within the capability of chimpanzees – however, we only found evidence for such copying in one subject (Baluku), which is a proof-of-principle finding. As in Study 1, Baluku performed the target action after having seen the demonstrations (not before) – and again Baluku was the only subject to show copying behaviour. Taken together, Baluku’s data from Study 1 and 2 leave no doubt that he copied this particular familiar action (presenting).

Live-coding established only one subject (Baluku) as an apt performer of the target action (19 performances across Studies 1 and 2); this was later corroborated by video (reliability) coding: only Baluku was deemed to have performed target actions in Study 2. This likely means that E1’s live-judgements in all other cases (i.e., in Study 1) had been too generous.

However, even though Baluku could correctly use this familiar target action in a gestural context, he first learned to use it in a technical context (i.e., in Study 1). This raises the possible objection that he might not have copied the familiar action in the social context if Study 2 had been conducted before Study 1: i.e., his target actions in Study 2 might have been due to a carry-over effect from Study 1. But, the fact that Baluku did not show the target action in the very first (baseline) trial of Study 2 suggests that Baluku did not simply transfer the target action of Study 1 to all potentially rewarding contexts in general. Instead, our data suggests that Baluku truly copied the target action in a social setting via contextual imitation in both cases (Study 1 and 2), for in both cases he only used the target action after having seen it be performed by the demonstrator.

Even though Baluku’s copying was impressive, he was not required to copy a completely novel behaviour in either Study 1 or 2 (which would be production imitation; see introduction), since the target action was in his repertoire prior to both studies (i.e., contextual imitation). In the three remaining studies (Studies 3 to 5) we thus examined (one year after Study 1 and 2) whether Baluku’s apparently exceptional copying skills (or motivation) would also extend to the copying of novel actions, i.e. whether he would be able to show production imitation (to copy novel target actions outside his repertoire).

We concentrated on Baluku and refrained from testing the majority of our other earlier subjects again, partly due to time constraints and partly because Tomasello et al. [40] had already established that novel target actions are not generally copied by chimpanzees. We felt that Baluku could be regarded as a promising candidate who may break the pattern observed by Tomasello et al. His performance in the contextual imitation domain might have been indicative of him being a special case for copying in the production imitation domain.

However, even assuming Baluku would prove able to copy novel actions, the question of whether Baluku only copied in the social context of Study 2 because of a carry-over effect from the non-begging context of Study 1 would remain somewhat unresolved. To address this issue we presented Baluku with the novel target action in a social context first, rather than beginning with a non-begging context as we have done for the familiar actions (i.e. Study 1).

Prior to Studies 3 to 5, we trained our demonstrator (Mawa) to perform a novel action (chimpanzee praying). If Baluku’s copying in Study 2 had nothing to do with an order effect and, if Baluku was able (and motivated) to copy novel actions, then he should copy in this setup just as he did in the previous ones.

## Study 3 Chimpanzee Praying as Begging

### Methods

#### Subjects

Baluku was the only chimpanzee to show evidence for the copying of target actions in Studies 1 and 2; only he was used therefore as a subject in Study 3 (see table 3).

### General Procedure

**Figure 4:**
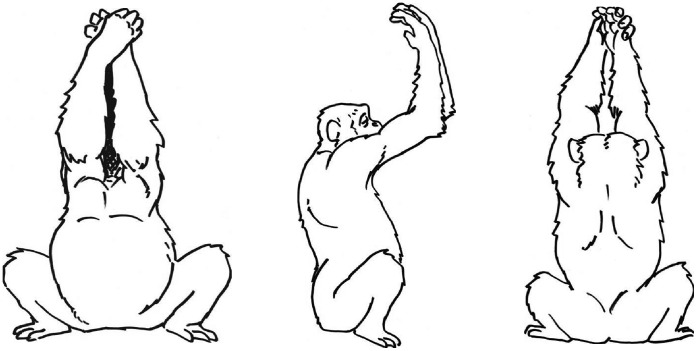
Schematic drawings of the target action chimpanzee praying; used in Study 3, 4 and 5.

For Studies 3 to 5 Mawa was trained to perform a different target action than before: chimpanzee praying. Here, Mawa was trained to squat and to raise both arms so that his biceps were perpendicular to his chest and his forearms were lifted in parallel to his erect torso. Finally, his hands had to be placed one over the other with both palms facing his head (see Fig. 4). Mawa was trained by way of clicker-training and molding to perform the chimpanzee praying gesture (see Fig. 4; see above), an action that is not within the chimpanzee repertoire.

Study 3 to 5 took place one year after Study 2. Study 3 resembled the full demonstration condition of Study 2 in all respects except that on the first two days of testing Baluku only received one baseline trial per day. From day three, Baluku received the first demonstrations of the novel target action (demonstration phase). Baluku was tested on four days during the demonstration phase. On each day of the demonstration phase Baluku received two demonstration sessions (ten demonstrations each) - each followed by one trial. Thus, overall eight demonstration trials were conducted with Baluku (and ten trials in total when including the two baseline trials).

### Results

Baluku did not perform the target action (chimpanzee praying) in the baseline trials. Later, Baluku was attentive to demonstrations, observing at least one demonstration per testing day. However, he did not perform the target action in the experimental trials. Thus, we found no evidence that Baluku had copied the novel target action. Instead, Baluku performed general begging actions, including both *normal* chimpanzee begging gestures (like lip-extended, arm/foot out of the cage) – as well as the target action of Studies 1 and 2 (i.e., presenting).

### Conclusions

Here we replicated the original gesture copying study by Tomasello et al. [40]. As in this original study, we trained a demonstrator to perform a target action which was novel to the observer in a begging context. Our subject, Baluku, failed to copy this novel action in the gesture copying context, just like the subjects in Tomasello et al. [40] – despite the fact that our test subject here (Baluku) had proven unique in Studies 1 and 2 at copying a familiar target action. Thus, while Baluku showed evidence for contextual imitation (Study 1 and 2), he failed to show evidence of production imitation (Study 3).

Interestingly, one year after Study 2, Baluku still used the begging gesture which he had learned in that earlier study (presenting). (According to the chimpanzee keepers present on the island at that date, Baluku performed the begging action presenting only to E1– starting at the time of Study 3). This means that he was generally still motivated by the task, but it also showed the carry-over effects of the earlier studies (be it due to conservatism or functional fixedness). But, contrary to Tomasello et al.’s [40] claims, our study showed that at least some non-enculturated chimpanzees can learn gestures observationally, but also that in this they may be restricted to contextual imitation, and may be unable (or unmotivated) to use production imitation. Thus, Tomasello et al.’s own criticism [40] of their study can no longer be strictly upheld: it was not the case that the begging context per se hindered chimpanzees from copying (conservatism, in other words, while perhaps a problem, did not hinder at least the addition of one action in one context: see results of Study 2) – the real problem seems to lie with copying novel actions instead.

Yet, a special version of this original critique could still be upheld. There remains the possibility that conservatism may not apply to all chimpanzees at all times. Maybe at least some chimpanzees can learn one and only one additional begging gesture to their normal repertoire – and (additionally) they may do so only if all external and internal factors are right. In our studies, this may have been the case for Baluku. In other words: having learned this single extra gesture (here: presenting), no other gesture may be added (except, perhaps yet another familiar action – not tested here). In yet other words, conservatism may represent a gradient, rather than being an all-or-nothing phenomenon.

Another potential explanation for Baluku’s failure to copy in Study 3 may have been the (intended) absence of a carry-over effect for this particular novel target action (chimpanzee praying). An alternative explanation for Baluku’s copying in the social context of Study 2 was a potential carry-over effect from the technical context of Study 1 and this prompted us to first test Baluku’s ability to copy a novel target action in the gestural context (i.e., Study 3). It still remains possible therefore that Baluku could have copied the novel action used in Study 3 if it had been demonstrated to him in a technical context. Thus, in Study 4, we tested Baluku once more with the same novel target action (chimpanzee praying) that we had used in Study 3– but this time we used the potentially easier technical context.

A final problem of Study 3 could potentially have been the initial baseline trials, in which Baluku might have felt obliged to try to use all general approaches that had previously secured him rewards – this may have activated his pre-acquired begging action presenting, which might then have interfered with his performance in later trials.

To move beyond these possible confounds, and to again test whether Baluku was actually able to copy a novel target action, we re-introduced a non-begging context for Study 4 (as used in Study 1), while retaining the novel target action chimpanzee praying. In order not to tap into potential conservatism, we also changed the context by altering the outer appearance of our apparatus. We called this new, changed, board the prayer-board, due to the type of novel action required to make it release a reward (i.e., chimpanzee praying; as used in Study 3). Furthermore, we did not include any baseline trials in this study, so that Baluku would not fall back into old behaviour patterns when the correct solution was yet to be presented to him. Additionally, in all of Baluku’s earlier baseline trials he (or indeed any other subject) had never shown any action even remotely resembling the chimpanzee praying gesture, and indeed this was true of their general behaviour outside testing. And so a baseline was not even strictly necessary anymore (i.e., earlier baseline trials and earlier behaviour were sufficient to establish that this target action did not occur spontaneously in our kinds of settings).

## Study 4 Prayer-Board

### Methods

We re-used the peanut dispenser from Study 2, but changed the outer appearance of the wooden board part. We painted the board cream and replaced the red circle with a blue rectangle around the reward hole. Mawa was trained to perform his praying gesture while squatting in front of this prayer-board. Unlike the presenting-board study, Mawa’s action was not required to touch the board directly, for his praying actions were directed to a position on the mesh well above the prayer-board.

Testing lasted five days, with three trials per day. We did not implement any baseline trials – all trials were experimental. Baluku was tested alone as in Study 3, and in a way just as the subjects in the full demonstration condition of Study 1– with the only other exception that in this case all trials lasted 5 minutes and demonstrations were of the target action chimpanzee praying.

### Results

Baluku was attentive to demonstrations and watched at least one demonstration per testing day. However he did not perform the novel target action. Instead, from day one onwards, he presented towards the prayer-board in the same way as he had done towards the presenting-board in Study 1.

It is also noteworthy that, during the training sessions of Mawa for the chimpanzee praying gesture, another male chimpanzee Asega (nine years old by then; who we never tested in the present studies) was present at all times (sometimes even in the same room). He thus saw Mawa perform countless rewarded instances of the chimpanzee praying action, but Asega never even showed approximations of this action himself during or after the training.

### Conclusions

Baluku did not show the novel target action (chimpanzee praying), even though the learning context was a non-begging instead of a begging one. Thus, we can exclude the possibility that Baluku’s failure to copy in Study 3 was due to a solely social begging context. This leaves three possibilities. Firstly, Baluku might not be able (or willing) to copy novel actions in general (regardless of the type of context) – and this would then support a more general disability for production imitation in chimpanzees – since no subject in Tomasello et al. [40] was able to copy a novel action either. Or, secondly, the conservatism hypothesis put forward in Study 3 may hold also across contexts, i.e., even particularly talented and/or willing chimpanzees may only be able to copy one action for one particular context (where this would then include both social and non-begging ones). Lastly, chimpanzees might be able to copy more than one action for a particular context, but only if the actions are familiar to them already: in other words, they may show high flexibility in contextual, but not production imitation. While this possibility was not our focus and was thus not tested, it should be kept in mind here that Baluku was the only subject that imitated contextually at all in Study 1 and 2, suggesting such flexibility to be limited in the first place.

Since Baluku used the presenting action in this study (which he had already learned in Study 1 and Study 2), we cannot fully exclude any of these three possibilities. In fact, the extended conservatism theory is partly supported by our data by the very fact that Baluku re-used earlier behaviour patterns. In the next (and final) study, we enhanced our efforts to change the general context – and thereby hopefully overcoming possible conservatism effects – by changing our board apparatus more extensively.

In Study 5 we made a final attempt to remove previous contexts (and context-correlates) from our non-begging test situation: We were able to implement a change of context in several dimensions. For example, we made a completely new board, avoiding many resemblances to the previous two boards (i.e., those used in Studies 1 and 4). Furthermore, in Study 1 (unlike in Study 3) the target action physically connected/ended with the apparatus itself, and so we designed the new board in such a way that our novel target action physically connected/ended on it as well, which may increase copying by providing an outward behavioural anchor. We called our new board the mini prayer-board, owing to its small dimensions.

Finally, another potential difference between our novel target action studies (Studies 3 and 4) and our familiar target action studies (Studies 1 and 2) might have been that we tested Baluku alone in the later studies – a context which might have been detrimental to copying (but note that Baluku had performed the familiar target action in the extra reliability test of Study 1 even though he was then tested alone). Thus, in this study, we added two other subjects to the testing situation, which added both potential social support [56] as well as a competitive situation (*sensu* [57]) – and which incidentally became extra test subjects themselves.

## Study 5 Mini Prayer-Board

### Methods

Study 5 resembled Study 4 but with the following differences. The new board was much smaller than that used in Studies 1 and 4 (it was only 50 cm high and 20 cm wide – compare with previous dimensions of 27 cm high and 93 cm wide). Our new board was also installed at a different height compared to before (50 cm higher than in Studies 1 and 4). The board also had a novel pattern painted on it (it was painted green and with 5 mm thick blue wavy-lines drawn – with a permanent marker; 16 lines in total) from the left to the right. It was also installed in a different room (i.e., the neighbouring room). The size of the reward hole was reduced and now measured only 3×3 cm. In order to even change the three-dimensional structure of the board, another piece of green wood (with the same wavy patterning) was screwed into a position above the reward hole (this extra piece measured 20×20 cm). Finally, the whole mini-prayer-board was oriented upwards, rather than horizontally.

We ran five sessions, spread over three days – which meant that sometimes there were several sessions per day (first day: session 1 and 2; second day: session 3 and 4; third day: session 5. Yet we allowed at least one hour between sessions). We added two further subjects (randomly chosen) to the test situation in Study 5 (both males: Okech (six years old) and Bwambale (eight years old) – to provide for potential social support [58]). We could not add more subjects, again due to time constraints.

### Results

Attention levels were high for all three subjects. All subjects saw at least one demonstration on each test session. Yet, none of the three subjects, including Baluku, performed the novel target action (chimpanzee praying). Instead, Baluku repeatedly walked his feet backwards up the wall (while facing downwards) – apparently in order to present to the mini-prayer-board (immediately from trial 1 of session 1). The other two subjects (Okech and Bwambale) did not even perform the presenting action.

### Conclusions

Neither Baluku nor the two additional subjects, Okech and Bwambale, copied the novel target action. Thus, Baluku failed in three contexts to imitate productively (Studies 3 to 5) while he copied the familiar target action presenting with apparent ease in Studies 1 and 2 (i.e., he started copying the presenting action immediately after his first demonstrations in these first two studies). The lack of copying in Study 5 was not due to the lack of social support (or the lack of a competitive factor), since two extra subjects were incorporated into the situation. Also, the lack of copying was not due to the spatial orientation of the target action, since in Study 5 we had Mawa touch the mini-prayer-board with his arms during the demonstrations (just as Mawa’s back touched the presenting-board in Study 1). Overall, Baluku showed no evidence for copying of novel target actions across Studies 3 to 5.

Due to the fact that Baluku presented again in Study 5, we still cannot exclude the possibility of conservatism. Thus, as an alternative to the general inability to copy novel actions hypothesis, Baluku might have been fixated on presenting in a wide variety of contexts that resemble those of both Study 1 and Study 2. If this is the case, however, then it would mean that even Baluku’s copying ability is, overall, very restricted. It would mean that very few chimpanzees copy at all (i.e., in our study only Baluku) and that these few can only copy one (or more) familiar action(s) for a particular context – with the context being very broad. Subjects would then only have *one* chance to copy *one* novel action (for a broad context); namely when no other, familiar action, had already taken that place.

## Discussion

In line with the original findings of Tomasello et al. [40], we found no evidence for production imitation in chimpanzees. When using a familiar target action on the other hand, we did find evidence for copying (contextual imitation) – but only in one single male chimpanzee (Baluku). Both the findings of Tomasello et al., as well as our findings, suggest that novel action copying (production imitation) is outside the ability and/or motivation of non-enculturated chimpanzees. The same is probably true (at least for the majority of non-enculturated chimpanzees) when it comes to contextual imitation (in both non-begging and begging contexts). Yet, in some special cases, like the male Baluku in our study, single chimpanzees can and do copy actions – at least if they are familiar ones. In our studies, this special chimpanzee copied a familiar action in both a non-begging as well as a begging context (but his latter performance here may possible owe to carry-over effects of the earlier, non-begging context study).

One possibility for why Baluku failed to copy novel actions could be that our novel target action (chimpanzee praying) might have been too difficult to copy, not due to its novelty, but due to some other factor. For unknown reasons, the familiar target action (presenting) may be generally easier to copy – at least, easy enough for a gifted chimpanzee to copy (like Baluku). For example, our novel target action involved the precise movement of two limbs in space, whereas the familiar target action only required the movement of one body part. Future work could thus try a full test battery of actions with Baluku (in the way that it was done with human-raised and enculturated chimpanzees: [59]). This would however be a study that would require considerable time. However, in general we do not think that this type of critique applies in this case, for the following reason: In Tomasello et al [40] novel actions were used which were comparable in terms of general difficulty level to the familiar action used here (i.e., touching the mesh with the top of the head, instead of touching it with the back). Thus, Tomasello et al. established that novel actions are not copied by chimpanzees, even if they seem to be relatively easy. Thus, novelty rather than general difficulty appears to be the factor responsible for failure.

An alternative explanation for Baluku’s special case could be that negative carry-over effects might have been responsible for his failure to copy novel actions in Studies 3 to 5. In particular, Baluku’s failure to learn a novel action might have been due to him having already copied an extra action previously in both types of context (i.e., in Studies 1 and 2) – potentially blocking him from learning yet another (which would be a special case of conservatism best described as functional fixedness). In other words, it might be that Baluku only had one action slot vacant for each particular task/context, which had already been filled in Study 1 and 2. However, an explanation based on such hypothesized filled action slots seems unlikely for three reasons: 1. Baluku’s failure to copy novel actions is in full agreement with the original findings of Tomasello et al. [40], where all subjects tested failed to copy novel actions despite not having had the previous experience that Baluku had (i.e., the same type of conservatism could not have been responsible for the negative findings of Tomasello et al.); 2. Additionally, in Study 5 neither Bwambale nor Okech (nor Asega during the training sessions of our demonstrator) learned the novel action, even though they (like the subjects in Tomasello et al.) had not previously acquired any alternative action to solve this problem; 3. There is currently no evidence that any unenculturated chimpanzee has ever convincingly copied a novel action. Some go even as far as to suggest that this is even true of enculturated and/or trained apes in so-called Do-As-I-Do studies; see Byrne & Tanner [19] – though this conclusion may hinge more on the definition of what exactly constitutes a novel action. What is clear is that enculturated and/or trained apes are better at action copying than non-enculturated apes are. In conclusion, neither Baluku nor other chimpanzees seem to copy novel actions. This might be due to either a lack of skill or to a lack of motivation – yet the fact that Baluku copied (familiar actions) in Studies 1 and 2 suggests to us that the problem is not one of general motivation – but rather one of skill. Similarly, Byrne & Tanner found no evidence for novel action copying in a gorilla that seemed motivated to copy familiar actions (even without being – at least at the time of testing – rewarded to do so).

Baluku’s failure to copy cannot be due to a general impossibility to learn more begging gestures once a chimpanzee is already able to perform some – since Baluku has indeed shown evidence of adding a (known) gesture from a different context to his begging gesture repertoire in Study 2. Thus, we have been able to show that the begging gesture context does not always exclude the learning of new begging gestures in chimpanzees (which was the critique raised against the original study of Tomasello et al. [40]). At the very least this critique cannot be valid for all types of actions and/or for all chimpanzees. Instead, in order to copy a begging gesture chimpanzees need to be especially motivated and/or able (i.e., like Baluku) and the target action might need to be a familiar one.

Additionally, copying of the target action in a technical context might need to precede copying in a social context. Corroborating this view, in so-called Do-As-I-Do studies, well-trained (perhaps enculturated) apes perform much better when the target actions are “anchored” towards an object (see review in [37]). Thus, the technical setup in Study 1 may have provided a cognitive anchor for Baluku to copy the target action, which may have helped him transfer the very same target action to Study 2. Contrary to this hypothesis, Baluku also failed to copy the novel target action in Study 5, where the target action was indeed anchored on the apparatus.

Importantly, in one earlier study, it was also Baluku who had shown anecdotal copying behaviour of familiar actions [52], [60]. In that (nutcracking) study, three of the tested chimpanzees were claimed to have copied familiar actions (Baluku, Umugenzi and Ikuro). Marshall-Pescini and Whiten noticed that Baluku performed cracking actions *in the air* while watching the demonstrator nutcrack (i.e., Baluku performed what could be regarded as hitting actions and without having had a tool in his hand – directed to the ground instead of to a nut). Baluku performed this type of actions for a total of seven times in that study – suggesting that he might have copied them (though it should be noted that he had earlier attempted nutcracking himself, which means that the cracking actions were not new to him [60]). The behaviour of the other two subjects mentioned by Marshall-Pescini and Whiten [52] was much less convincing (and this study was also never designed to tests for such copying, which is why all these cases, including Baluku’s, remain anecdotal. Similar reasons prevent one from drawing strong conclusions from anecdotal observations (e.g [61]). Umugenzi only performed this type of action once. Ikuro did not perform this action at all – she only showed rocking motions with her whole body. However, Ikuro performs rocking motions frequently and in many contexts (pers. observation). In sum, Baluku’s performance across two studies set him apart from the rest of the subjects that were tested. Baluku seems to be truly special – which could be due to genetic and/or ontogenetic and/or social reasons. As for genetics, such an explanation can only be speculated at this point, since there is no current way of testing such a hypothesis (but it also cannot be excluded). This leaves ontogeny and social relationships open for further discussion.

As for social relationships, at the time of both of our studies Baluku had a close relationship with our demonstrator (Mawa): Baluku, as a younger male, tried to befriend Mawa. However, the same may be said of most young males that we tested – yet none of which copied. Thus, we have no evidence that special relationships matter in chimpanzee copying (in contrast to the claims of de Waal [61]). When we analyze across studies the sex (or species) from which Baluku copied, also no clear pattern emerges. In Marshall-Pescini & Whiten [52], Baluku showed anecdotal evidence for copying familiar actions both if these were performed by a male conspecific as well as by a female human experimenter (see also more detailed descriptions, as well as some discussion about the implications for mirror neuron research, in [60]). In our studies, we choose a male demonstrator (since in the original action copying study by Tomasello et al. [40] all demonstrators were females), but apparently this factor does not make a difference. Thus, we conclude that the gender and the species of the demonstrator do not have any (significant) effect – and neither does their relationship.

This leaves ontogeny. Baluku may not be very special in comparison to his island companions. The only potential ontogenetical difference between Baluku and the other chimpanzees is that Baluku had to be taken care of twice instead of once by humans: the second care session was necessary because he was severely bitten by his group mates and would not have survived on his own. However, on both occasions he was treated by the human keepers as a chimpanzee, and not as a human (in general, care is taken at Ngamba Island, and by cooperating partners, that during times of unavoidable care the chimpanzees are treated as chimpanzees, so as not to enculturate them in any way (Debby Cox, pers. comm.)). Baluku’s injuries resulted in one severely maimed hand, potentially the most important developmental difference between him and his fellow chimpanzees. Indeed, in the problem solving study by Horner & Whiten [62] Baluku developed a different solution technique to the rest of the chimpanzees, probably due to his left hand being injured which prevented him from using the same actions as the rest of the subjects. Potentially, therefore Baluku might be especially apt at copying due to his injury. He might have to rely on action copying skills more than other chimpanzees due to his being less versatile with tools than the other chimpanzees (i.e., emulation in his case might not work very well). However, this reasoning could also be flipped: It might likewise be said that Baluku should be less prone to action copying, since he cannot perform the same (manual) actions as the rest of his group. Whilst this may seem like a good ad hoc explanation for why he did not perform well when the target action required partly the use of his hands (i.e., Studies 3 to 5, chimpanzee praying, the novel target action) – it does not explain the anecdotal evidence for manual hammering actions in Marshall-Pescini & Whiten’s nutcracking study [52]. Currently, therefore, a hypothesis based on his injuries remains highly speculative.

To summarize, Baluku’s copying, in contrast to the performance of all other chimpanzees tested, may be a result of Baluku being especially talented or because of a special ontogeny (injuries?) or a combination of both. Currently we are unable to pinpoint the main reason for Baluku’s superior performance. We hope that future studies performed elsewhere (Baluku himself largely ceased to be a suitable subject, since our data suggests that he would be showing carry-over effects for a long time and across contexts), using the same methodological setup as our study, might help identify more chimpanzees who copy and bring us closer to detecting some of the underlying patterns (genetically and/or ontogenetically) that affect the likelihood of whether a chimpanzee will copy or not.

Our study supports the idea that non-enculturated chimpanzees generally do not (or even cannot) copy novel actions – be it in non-begging or in begging contexts [2], [17]. Together with the apparent rareness of copying of familiar actions (Study 1 and Study 2) our results suggest the following: Chimpanzees do not readily action copy in general (based also on a literature review; [2]). It takes special situations and/or subjects in order even to copy familiar actions (contextual imitation), which renders the evolution of culture in chimpanzees a difficult, and highly unlikely process (especially given the aforementioned added detrimental effects of conservatism and functional fixedness). This data would suggest that non-enculturated chimpanzees could not sustain a culture based, even to a small degree, on the copying of actions. In line with this view, recent work suggests that action copying is unlikely to be a major underlying acquisition mechanism for ape gestures (chimpanzees: [40]; gorillas: [63]) and also that genetic predispositions may play a larger role in explaining chimpanzee cultures than was previously thought [64] – the latter simultaneously supporting our earlier and complementary hypothesis (the zone of latent solutions hypothesis; [2]).

Recently Hobaiter & Byrne [65], presented data on the social transmission of liana scratching techniques in wild chimpanzees, which they claim to be based on action copying and which therefore goes against our prediction. However, the transmission of scratching might not even have been based on action copying, since these behaviours were object-centred (i.e., towards the lianas in conjunction with body parts) and which allows for other social learning mechanisms to underlie such transmission (notably result copying). Supposing that the observed spread had been based on action copying, the underlying actions were probably not novel ones to the chimpanzees – a minimal interpretation also shared by Hobaiter & Byrne [65] themselves. Thus, these chimpanzees would have copied only familiar actions from each other. In that case our hypothesis might merely be too strong, and chimpanzees could develop cultures among themselves based on action copying – but only as long as these actions are familiar to them. Yet, we would still not expect any culture consisting of the copying of novel actions.

This lack of action copying among apes has significant implications for the kind of cultures that chimpanzees (and potentially apes in general) can sustain. In the following we will argue that this hypothesis may also explain the relative lack of cumulative culture in apes [2]. If not enough chimpanzees in a group are willing and/or able to copy actions, then the retention rate of many types of innovations that are somewhat based on innovated actions will not be large enough to sustain these innovations across generations, or even to let them spread through the current generation. In particular, any such innovations that are unlikely to be readily invented by other, naïve individuals, will then die out together with the innovator – since the only possible transmission system (hi-fi copying  =  action copying) – cannot in all likelihood happen. Thus, whereas humans do copy whole behaviours (i.e., the details of the cultural design, including fine-tuned actions, goals and results), apes in general copy observed results instead. Why do they do this? We believe they do this because it suffices for them. There is no behavioural tradition in wild apes, we postulate, that could not be invented by normally developing individual apes – given the right motivation and materials (and here some social learning certainly helps, e.g. if their focus is drawn to certain parts of their environment by others (different forms of socially transmitted enhancement) – and thus social learning can still play a crucial role in explaining frequencies of behaviours across populations. What social learning in great apes does not explain – in our view – are the actual forms that these behaviours take). In other words, apes constantly *re-invent the wheel* – and the general type of wheel they produce is the only one they use and need [2]. The learning mechanism best fitted for such transmission is (results-based) emulation learning, based on genetically transmitted (not culturally transmitted, as is additionally the case in humans) problem solving skills of the species [2], [66], [67] or even subspecies (see [64]).

It might be objected that recent findings point to some ability of apes to produce cumulative culture after all. Sanz et al. have interpreted some data on the manufacture and use of brush-tipped fishing probes in chimpanzees in the Goualougo Triangle, Republic of Congo in this way [68]. The behaviour involves the manufacture of certain herb-stem tools (prior to their use, even) that are given frayed ends (mostly by pulling them through the teeth), a design feature which may increase the effectiveness of the tool in gathering termites. In its strictest form, the ZLS hypothesis [2] would predict however that this behaviour should not need to be transmitted. Individuals should be capable of inventing it (Sanz et al. embrace parts of this logic when they write: “The absence of this behaviour in several other chimpanzee populations suggests that this is a skill acquired during ontogeny, not necessarily a species-specific trait.”). If the behaviour appears elsewhere, therefore, it would cease to be a case of cumulative culture. In practice there are two ways that this could be shown: either the necessary material is provided to individuals who are naïve to the technique and where at least some show the behaviour over time – without the help of a cultural history (see [69], [70]), or other (unconnected) populations can be found after all which show similar behaviour patterns. Both methods would show that the behaviour in question developed in a specific form regardless of cultural history (thereby exemplifying underlying constraints and channelling). The first method has got the additional advantage that it is potentially able to show that the behaviour does not require multiple generations to develop. In the case of brush-tools, no experimental study is yet available. As for natural experiments, currently there exist no other reports of such a behaviour elsewhere (other brush-tools found in chimpanzees are most likely the result of unintentional object manipulations, [68]; though see [71] for some first (though indirect) evidence for intentional frayed-tool usage (if not also production) in an unconnected population in Loango National Park, Gabon). However, not much time has passed since the analysis of Sanz et al., and it can sometimes take several decades before a behaviour found in one population is also found in others (e.g. nutcracking, long thought to be restricted to populations in western Africa, has now also been found eastwards of it –1700 km eastwards – in an unconnected population [72]). The same could happen to the case of brush-tipped fishing probes (or the behaviour may appear in captive latent solution experiments). For these reasons - for the time being - we reserve our judgement with regard to cumulative culture in chimpanzees although we concede that brush-tipped fishing probes are the best potential case known to date.

Human culture has accumulated beyond the spontaneous grasp of naïve human individuals. The reason for this is that accumulated culture includes behaviours and artefacts (subsumable under a general design criterion) that are arbitrary, yet highly complex, from a naïve perspective (e.g., technical solutions may become *cognitively opaque* [3]; see also introduction). In other words, this accumulated design could not be independently invented by a culture-naïve individual from scratch [73], [74], [75] as, e.g., a multivitamin pill or Salsa dancing. It thus has become improbable in design for anyone outside the current cultural line of transmission and as such it must be copied or otherwise it cannot be acquired.

We have argued that one of the most important learning mechanisms for cultural design is action copying. For this view we have several reasons: First, accumulated culture can sometimes be entirely based in action – as for example in dance, or in sign language – and so does not even involve artefacts (for which emulation learning could potentially suffice). Obviously, in this case only action copying will allow for successful transmission; especially since such cultures tend to increase arbitrariness over time (hence they become improbable to be invented individually; [2]). Second, even when understanding artefacts is required or at least possible the complexity of the learning can be substantially lessened if action copying accompanies results copying [10]. Third, all transmission processes are error prone, and one way to overcome this problem is to involve redundancy (redundancy is used where precise transmission is important, e.g., in the genetic code of the DNA there are two complimentary streams of code rather than just one). Action copying, if combined with results copying, results in redundant information. A third type of information can add yet another layer of redundancy: goal information (see [32]). These three sources of redundancy render the human transmission system much less prone to error than the single-stream ape system – i.e., one which may only be based on results copying ([18], but see [24]). And errors in transmission are in effect obstacles to accumulation [4]. Fourth, using different information streams allows for the successful transmission of very subtle cultural differences by utilising all available information streams at once and in combination (i.e., goals, actions and results). This can have a significant impact on the general efficiency (or adaptedness) of the resulting cultural design – it may especially render the human cultural system much more dynamic than *single-stream* systems. For example: Sometimes the goals might change (e.g., pretending to pick up the phone – to end a conversation). Sometimes the action may change (e.g., squeezing grapes with the feet instead of with the hand – as in wine-making) and sometimes the result may change (e.g., using a hammer as a door block). In theory, all information channels can carry new information independently of one another and so the more information channels one has, the more directions and details of design are possible.

The fact that in this study we have identified one non-enculturated chimpanzee (for a case study in a different species – gorillas – see [19]) who can spontaneously copy familiar actions is informative for possible evolutionary scenarios. While we think that current human culture would not be sustainable purely by way of contextual imitation (and this is a claim that is preliminary, given that usually human imitation studies pay less attention to the distinction between novel and familiar action copying than do ape studies) – a less complex culture (with less complex cultural variants) might. A culture based on familiar actions could already go beyond the types of traditions that apes sustain (i.e., beyond non-imitation based traditions, see [2]); in other words, populations made up entirely of subjects as capable as Baluku could potentially produce and sustain cultures that go well beyond what is currently observed in wild ape populations. Based on the superior performance of Baluku, such a community could one day be found (or may already have been found [65]). As long as abilities such as Baluku’s could potentially spread within a group (we suspect firstly via genetic means), a plausible evolutionary scenario could unfold, in which individuals begin initially to copy familiar actions – and eventually start to develop the ability to copy novel actions as well. Something along these lines seems to have happened at some point in the human lineage.

Finally, as mentioned in our introduction, humans use immediate action copying also as some kind of social glue – where imitation can help establish and maintain social relationships. Our study – with its general finding of a lack of action copying in chimpanzees – may thus also be taken to also suggest a lack of this kind of imitation in chimpanzees.

## Supporting Information

Figure S1 **Screenshot of the full demonstration condition of Study 1 (taken during the demonstrations).**
(JPG)

Figure S2 **Screenshot of the baseline condition of Study 1 (taken during the pseudo-demonstrations).**
(JPG)

Video S1 **Baluku’s performance during reliability tests.**
(MP4)
