# Supplementary figures and images for: Untrained Chimpanzees (Pan troglodytes schweinfurthii) Fail to Imitate Novel Actions
Source: PLoS One. 2012 Aug 8;7(8):e41548. doi: 10.1371/journal.pone.0041548 (PMC3414512; doi:10.1371/journal.pone.0041548)

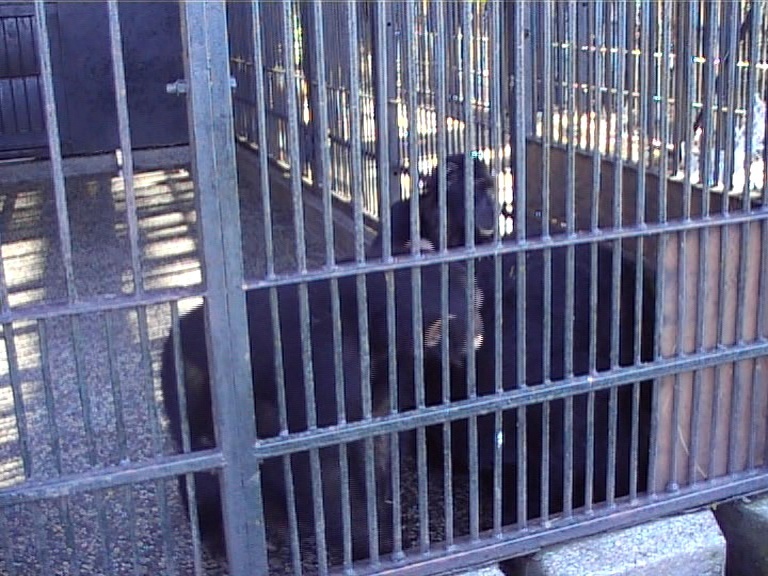

Supplement: Figure S1 — Screenshot of the full demonstration condition of Study 1 (taken during the demonstrations). (JPG) [file pone.0041548.s001.jpg]

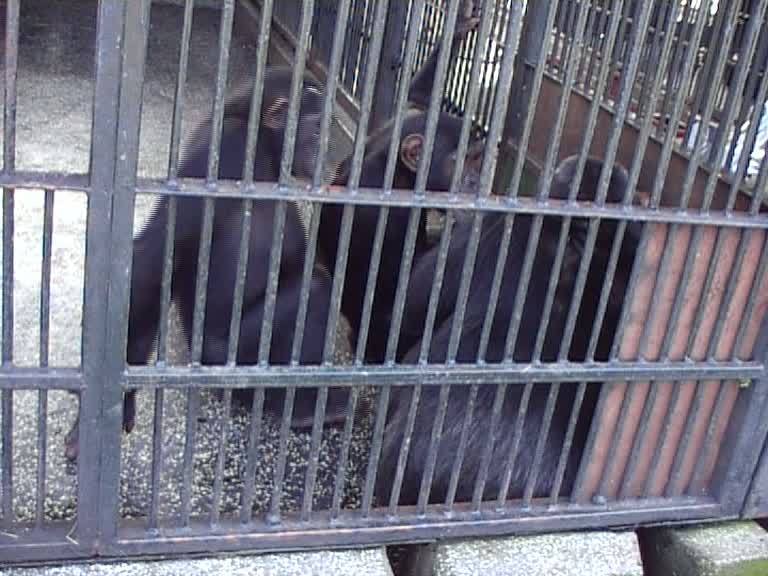

Supplement: Figure S2 — Screenshot of the baseline condition of Study 1 (taken during the pseudo-demonstrations). (JPG) [file pone.0041548.s002.jpg]
